# Effect of Microencapsulated Basil Extract on Cream Cheese Quality and Stability

**DOI:** 10.3390/molecules28083305

**Published:** 2023-04-07

**Authors:** Liliana Popescu, Daniela Cojocari, Ildiko Lung, Irina Kacso, Alexandra Ciorîţă, Aliona Ghendov-Mosanu, Greta Balan, Adela Pintea, Rodica Sturza

**Affiliations:** 1 Faculty of Food Technology, Technical University of Moldova, 9/9 Studentilor Street, MD-2045 Chisinau, Moldova; 2 Department of Preventive Medicine, “Nicolae Testemitanu” State University of Medicine and Pharmacy, 165 Stefan cel Mare Boulevard., MD-2004 Chisinau, Moldova; 3 Department of Physics of Nanostructured Systems, National Institute for Research and Development of Isotopic and Molecular Technologies, 400293 Cluj-Napoca, Romania; 4 Faculty of Biology and Geology, Babes-Bolyai University, 5-7 Clinicilor Street, 400006 Cluj-Napoca, Romania; 5 Faculty of Veterinary Medicine, University of Agricultural Sciences and Veterinary Medicine, 3-5 Calea Manastus Street, 400374 Cluj-Napoca, Romania

**Keywords:** microencapsulated basil extract, antimicrobial activity, cream cheese, quality, shelf life

## Abstract

The antimicrobial and antioxidant effects of plant extracts are well known, but their use is limited because they affect the physicochemical and sensory characteristics of products. Encapsulation presents an option to limit or prevent these changes. The paper presents the composition of individual polyphenols (HPLC–DAD-ESI-MS) from basil (*Ocimum basilicum* L.) extracts (BE), and their antioxidant activity and inhibitory effects against strains of *Staphylococcus aureus*, *Geobacillus stearothermophilus*, *Bacillus cereus*, *Candida albicans*, *Enterococcus faecalis*, *Escherichia coli*, and *Salmonella Abony*. The BE was encapsulated in sodium alginate (Alg) using the drop technique. The encapsulation efficiency of microencapsulated basil extract (MBE) was 78.59 ± 0.01%. SEM and FTIR analyses demonstrated the morphological aspect of the microcapsules and the existence of weak physical interactions between the components. Sensory, physicochemical and textural properties of MBE-fortified cream cheese were evaluated over a 28-day storage time at 4 °C. In the optimal concentration range of 0.6–0.9% (*w*/*w*) MBE, we determined the inhibition of the post-fermentation process and the improvement in the degree of water retention. This led to the improvement of the textural parameters of the cream cheese, contributing to the extension of the shelf life of the product by 7 days.

## 1. Introduction

A growing demand for safe, natural, and synthetic preservative-free food products leads researchers to develop new alternatives and sustainable approaches for food preservation [1]. Cheeses are dairy products widely consumed throughout the world as part of a regular diet, and are valued for their high content of proteins, fats, mineral (especially calcium), and vitamins [2]. Cream cheese is a fresh soft cheese used as an ingredient in many food applications [3]. Due to the high moisture content and a favorable pH, cream cheese is considered an optimal environment for the growth of pathogenic and spoilage microorganisms [4]. The addition of preservatives is one of the most used methods of ensuring the antimicrobial stability of cheeses [5]. Effective replacement of preservatives (e.g., sorbates, nitrites, etc.) is the focus of various recently reviewed studies [6]. Phenolic extracts from aromatic plants have attracted the attention of the scientific community regarding their safety as natural ingredients as well as their wide application in the food industry [5,7,8]. Basil extract is characterized by high antioxidant and antimicrobial activity [9,10], contributing to reducing the population of pathogenic microorganisms and to the expansion of the shelf life of perishable food products [11]. Direct addition of aromatic plants to food products is the most common method applied in the industry [12]. However, their use is still limited in food products because they can negatively affect the sensory and physicochemical characteristics of the products [13], interact with the components in food matrix [14], and are unstable to variations in pH, temperature, the presence of light, etc. [15]. Moreover, the utility of bioactive compounds is, indispensably, related to their bioavailability [16]. Microencapsulation is considered an effective technology that ensures higher stability of bioactive compounds during the manufacture of cheeses, preserving their antioxidant and antimicrobial activity throughout the products’ shelf life [17,18]. Recently, the functional activity of bioactive compounds from plant extracts encapsulated in various dairy products, especially cheeses and yogurts, has been reported [19,20,21,22]. Therefore, the objective of this work was to evaluate the antioxidant and antimicrobial activity of basil (*Ocimum basilicum* L.) extract, the efficiency of its microencapsulation, as well as the effect of the addition of microencapsulated basil extract on the sensory, physicochemical, and textural properties of cream cheese during its shelf life.

## 2. Results and Discussion

### 2.1. Total Polyphenol Content and Antioxidant Activity of Basil Extract

The total polyphenol content (TPC) determined from the basil (*Ocimum basilicum* L.) extract (BE) was 26.18 ± 0.21 mg gallic acid equivalent (GAE)/g dry weight (DW). Comparing the result obtained with the one from the literature, it was found that they are comparable. Thus, Nguyen et al. [23] determined that in basil leaves, there was a TPC of 29.60 mg GAE/g DW. The TPC, depending on the varieties and the part of plant from which the extract was obtained, varied between 2.30–7.11 mg GAE/g fresh weight (FW) [24]. Furthermore, the TPC from basil collected from different regions of the world varied between 2086–25,593 mg GAE/100 g DW [25].

Individual polyphenolic compounds in BE were identified using high-performance liquid chromatography equipped with a photodiode-array-detection-mass (HPLC–DAD-ESI-MS) method (Table 1). The antioxidant activity of BE was evaluated using two different spectrophotometric methods: DPPH (2,2-diphenyl-1-picrylhydrazyl) and ABTS (2,2′-Azino-bis(3-ethylbenzthiazoline-6-sulfonic acid)).

As shown in Table 1, a total of nine phenolic compounds were detected in BE. The compounds were assigned to phenolic acids (methyl-rosmarinate, rosmarinic acid, rosmadial, carnosol, dehydrodiferulic acid, and chicoric acid) and to flavonoids (luteolin-glucoside, querectin-rutinoside, and epigallocatechin). In terms of quantification, methyl-rosmarinate and rosmarinic acid proved to be the most abundant in the studied BE (17.08 mg/g DW and 13.81 mg/g DW respectively). The results obtained in this study are in agreement with other studies [26,27,28], where rosmarinic acid is reported to be the most represented phenolic acid in basil. BE showed chicoric acid and dehydrodiferulic acid values of 1.3 mg/g DW and 3.1 mg/g DW, respectively. Ghasemzadeh et al. [29] identified that the ferulic acid content was 3.29 mg/100 g dry material in sweet basil leaf extracts. Khatib et al. [28] reported higher values of chicoric acid in Moroccan sweet basil (9.1 mg/g dry extract) and a lower content of carnasol (3.2 mg/g dry extract) compared to our results.

Regarding flavonoids, luteolin-glucoside (0.85 mg/g DW), querectin-rutinoside (0.75 mg/g DW), and epigallocatechin (0.72 mg/g DW) were detected in BE. The content of quercetin and catechin in sweet basil leaf extracts reported in studies performed by Ghasemzadeh et al. [29] was 2.73 and 2.61 mg/100 g dry material, respectively; luteolin was not identified. Discrepancies between the levels of phenolic compounds in basil extracts reported in the literature may occur due to different cultivation and post-harvest conditions, as well as different cultivars and methods of obtaining BE. 

The content of total and individual polyphenols of BE correlated with its antioxidant activity. The antioxidant capacity of the BE determined using DPPH and ABTS methods was 644.75 mM Trolox/g DW and 8.95 mM Trolox/g DW respectively. Other authors found 19.91 mM Trolox/g DW in 80% methanol basil extract with the DPPH method [30], 162.16 mM Trolox/g DW in water basil extract using the DPPH method [28], 492.22 mM Trolox/g extract in 96% ethanol basil extract using the DPPH method, and 541.16 mM Trolox/g extract using the ABTS method [31].

### 2.2. Antibacterial Activity of Basil Extract

Originally, the in vitro antibacterial activity of BE against microorganisms was qualitatively assessed by the presence or absence of zones of inhibition. The extract exhibited antibacterial activity against gram-positive bacteria, gram-negative bacteria, and yeast. A potent inhibitory effect was demonstrated against strains of *Staphylococcus aureus*, *Geobacillus stearothermophilus*, *Bacillus cereus*, and *Candida albicans*, with diameters of inhibition zones ranging from 11.0 to 26.3 mm, as shown in Table 2. A lower inhibitory effect was recorded against *Enterococcus faecalis*, *Escherichia coli*, and *Salmonella Abony* strains, with the diameter of the zone of inhibition equaling 8.3 mm.

The results of the study on the minimum inhibitory concentration (MIC) and minimum bactericidal concentration (MBC) values of the BE are presented in Table 2. The MIC for strains of *Staphylococcus aureus*, *Geobacillus stearothermophilus*, and *Acinetobacter baumannii* was 11.2 mg/mL, and for *Bacillus cereus*, *Escherichia coli*, and *Candida albicans*, the MIC was 22.5 mg/mL. Antimicrobial inhibitory activity in higher concentrations was recorded on strains of *Enterococcus faecalis* and *Salmonella Abony*—45.0 mg/mL.

The BE showed a bactericidal and fungicidal effect at a concentration of 22.5 mg/mL on the bacteria *Bacillus cereus*, *Geobacillus stearothermophilus,* and the yeast *Candida albicans*. The bacteria *Staphylococcus aureus*, *Escherichia coli*, *Salmonella Abony*, and *Acinetobacter baumannii* were killed at concentrations of 45.0 mg/mL, and *Enterococcus faecalis* was killed at 90.0 mg/mL. Phenolic compounds present in BE can damage the permeability of the bacterial membrane through mechanisms such as substrate complexing, membrane disruption, enzyme inactivation, and metal chelation, resulting in ion leakage that can cause a loss of membrane proton motive force, leading to bacterial death. The blind control did not inhibit the growth of the tested bacteria [32].

Currently, among the most known and studied mechanisms regarding the action of antimicrobial agents are a wide variety of bacterial targets and processes, such as inhibition of protein synthesis, inhibition of metabolic pathways, interference with cell wall synthesis, inhibition of deoxyribonucleic acid (DNA) and ribonucleic acid (RNA) synthesis, and also the lysis of the bacterial membrane. The most common and studied mechanisms of bacterial resistance to antibacterial agents are antibiotic modification by enzymes, antibiotic inactivation, and expression of efflux pumps [33].

Various studies have demonstrated and reported that many plant extracts have antimicrobial properties [34,35,36]. Plant extracts, due to their antibacterial, antifungal, antioxidant, and anticarcinogenic properties, can be used as natural additives in many foods, and the use of plant extracts can improve food safety and overall microbial quality [37,38].

In this study, the BE exhibited potential activity against some of the representative foodborne pathogenic bacteria, such as *Bacillus cereus*, *Staphylococcus aureus*, *Escherichia coli*, and *Salmonella Abony*. Antibacterial activities of plant extracts have also been reported in other studies [39,40].

The reported results from different studies are difficult to compare, probably because of the different methods of testing bacterial strains and sources of antimicrobial samples used. A previous study showed that the methanolic extract of *Ocimum basilicum* L. from Turkey weakly inhibited the growth of bacterial strains from the genera *Bacillus*, *Escherichia*, and *Staphylococcus*, with inhibition zones of 7–12 mm, while only *Acinetobacter* was strongly inhibited (17 mm) [41].

### 2.3. Microencapsulated Basil Extract Characterization

**Figure 1:**
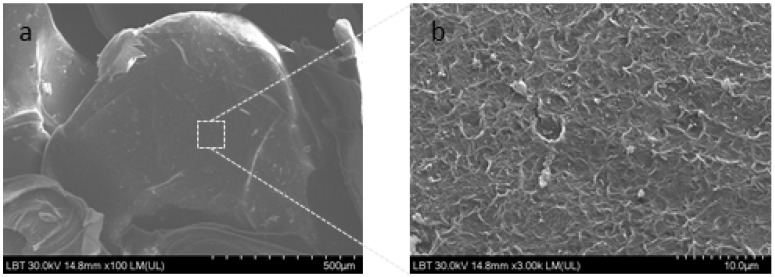
SEM micrographs of the microencapsulated basil extract sample (**a**,**b**).

The basil extract was encapsulated in sodium alginate (Alg) using the drop technique. The physicochemical parameters of the obtained microencapsulated basil extract (MBE) were moisture—6.21 ± 0.08%, swelling index—87.4 ± 0.4%, and solubility—22.1 ± 1.1%. The scanning electron microscope (SEM) images of the MBE are presented in Figure 1.

SEM analysis showed that MBE had a rough surface. The microcapsules were deformed during SEM measurements due to the presence of a vacuum, but an estimation of their size was attempted, and it was observed that their average size is around 1.08 ± 0.42 mm/0.81 ± 0.13 mm. Similar results were also obtained by other authors [42,43]. 

**Figure 2:**
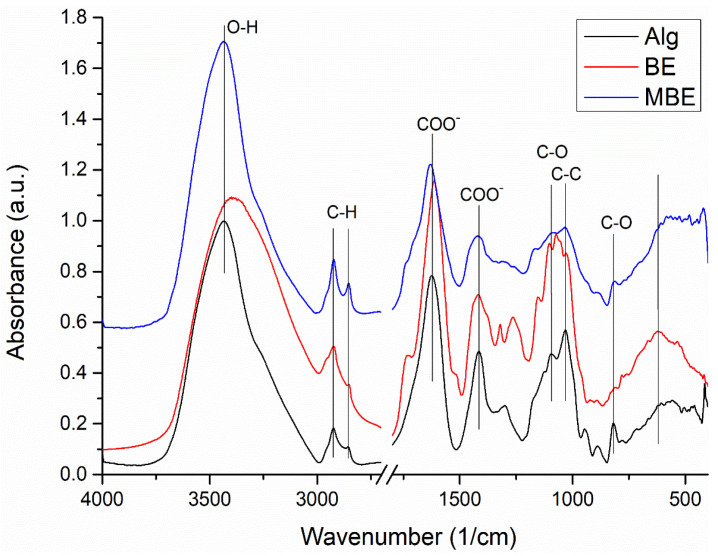
The FTIR spectra of sodium alginate (Alg), basil extract (BE) and microencapsulated basil extract (MBE), 4000–400 cm^−1^ spectral domain, 2700–1800 cm^−1^ split.

The characteristic absorption peaks of Alg (Figure 2) can be assigned as follow: 3434 cm^−1^ (stretching vibrations of -OH groups), 2924 and 2855 cm^−1^ (asym. and sym. stretching peaks of CH_2_ groups), 1624 and 1416 cm^−1^ (asym. and sym. stretching peaks of COO- salt groups), 1301 cm^−1^ (C–O stretching), 1173 and 1124 cm^−1^ (C–C stretching), 1095 and 1031 cm^−1^ (stretching of groups C–O and C–O–C in mannuronic and guluronic units, respectively) [44], 946 cm^−1^ (C–O stretching of the pyranosyl ring and C–O stretching with contributions from C–C–H and C–O–H deformation), and 818 cm^−1^ (C–O vibration of groups in α-configuration of the glucuronic units) [45].

The Fourier-Transform-Infrared (FTIR) spectrum of BE (Figure 2) showed the characteristic vibrational bands at 3395 cm^−1^ (phenolic O-H), 2923 and 2851 cm^−1^ (alkane C–H), 1728 cm^−1^ (carbonyl -C=O), 1615 cm^−1^ (alkene -C=C-), 1518 cm^−1^ (aromatic -C=C-), 1377 cm^−1^ (alkane -C–H), 1265 cm^−1^ (ether C–O–C), 1154 and 1103 cm^−1^ (alcohol -C–O), and 622 cm^−1^ (C–Cl) [46].

The FTIR spectrum of MBE (Figure 2) shows a wider absorption bands of lower intensity, but the characteristic vibrational bands of both components can be found in the spectrum, slightly shifted. Thus, the -C=O stretching is shifted from 1728 cm^−1^ to 1740 cm^−1^, and the -C=C- of extract and -COO- vibration of alginate shifted from 1615 cm^−1^ and 1624 cm^−1^, respectively, to 1629 cm^−1^. The vibrational bands of BE from 1103 and 1072 cm^−1^ were shifted and appear as a broad band with a maxima at 1086 cm^−1^.

The changes identified in the MBE mixture spectrum compared to the spectra of its components, Alg and BE, can be attributed to the existence of weak physical interactions between the components. Therefore, by encapsulating basil extract with sodium alginate as the coating material, the formed microcapsules can serve as polyphenol carriers for food.

The encapsulation efficiency of MBE was 78.59 ± 0.01%, which is considered high efficiency. According to the studies carried out by Tomé et al. [47], the encapsulation efficiency of the hydroethanolic extracts of basil, parsley, rosemary, thyme, and chervil varied from 68.24% to 93.39%, with higher results for rosemary. The high encapsulation efficiency of MBE demonstrates the formation of stable interactions between the reactive sites of Alg and BE.

### 2.4. Cream Cheese Fortified with Microencapsulated Basil Extract Characterization

#### 2.4.1. Physicochemical Analysis of the Cream Cheese Fortified with Microencapsulated Basil Extract

The physicochemical parameters of cream cheese fortified with MBE on the first day of storage are presented in Table 3.

Physicochemical parameters of cream cheese were not insignificantly influenced by the addition of MBE. Increasing MBE concentration in cream cheese led to a slight decrease in protein and fat content in the ranges of 5.82–5.73% and 23.04–22.72%, respectively. The sodium alginate in the MBE composition led to the retention of free water and, respectively, to a crease in the dry matter content of the cream cheese samples. The positive effect of hydrocolloids on water retention in Labneh cheese fortified with alginate-encapsulated pepper extracts was also observed by Balabanova et al. [48]. A similar behavior was previously reported in cottage cheeses with microencapsulated fennel extract [20], soft cheese supplemented with encapsulated olive phenolic compounds [19], and ultrafiltered cheese with encapsulated plant phenolic extracts [21].

#### 2.4.2. Evolution of the Cream Cheese Fortified with Microencapsulated Basil Extract Characteristics during Storage

The sensory properties of the cream cheese fortified with MBE, including appearance, texture, odor, taste, and overall acceptability, were evaluated during the 28-day storage time at 4 °C, and the results are shown in Table 4.

The appearance of the CC on the first day of storage was characterized by a homogeneous, slightly yellowish paste, without whey removal, in the case of cream cheese fortified with MBE—a uniform distribution of microcapsules in the cream cheese mass. All cream cheese samples were rated at 5.00 points. The texture of the CC, as well as that fortified with MBE on the first day of storage, was characterized by a fine, creamy paste. The addition of 1.2% MBE gave the cream cheese a lingering smell and taste of basil that intensified during storage, leading to a drop in the taste score to 4.20.

On the 21st day, the taste of CC and 0.3% CCMBE became slightly sourer, and as a result, the accumulated overall acceptance was 4.83 and 4.15 points, respectively. On the 28th day of storage, the texture of these samples became slightly softer, and at the taste level, a slightly rancid taste appeared as a result of the chemical and oxidative reactions of the lipids. In the case of the 0.6% CCMBE and 0.9% CCMBE samples, no changes in sensory quality were observed during the 28 days of storage. The addition of MBE at concentrations higher than 0.3% had a favorable impact on the storage stability of cream cheese compared to CC. This fact is due to the stability and gradual release of polyphenolic compounds from microcapsules with antioxidant and antimicrobial effect in cheeses [38].

According to the results of the sensory analysis, the optimal concentration of MBE in the cream cheese is in the range of 0.6–0.9%, an addition of 0.3% MBE to the cream cheese does not ensure sufficient stability of the product during storage, and when the concentration of MBE increases above 0.9%, a residual taste of basil appears even on the first day of storage. These findings are consistent with those of the study by Azarashkan et al. [49], in which it was shown that the fortification of ricotta cheese with nano-encapsulated broccoli sprout extract had no significant effect on the texture, color and smell of the cheeses on the first day. Research on the effect of the fortification of white soft cheese with white olive polyphenol capsules [19] and ultrafiltered cheese with white red beet, broccoli, and spinach leaf phenolic extracts encapsulated by complex coacervation [21] noted that the fortified samples obtained higher overall acceptance compared to the control sample.

The pH values of cream cheese fortified with MBE during the 28-day storage time at 4 °C, are shown in Table 5.

According to the data presented in Table 5, the addition of MBE slightly reduced the pH of the cheese cream samples analyzed on the first day of storage. CC had the highest pH, while the addition of MBE led to a decrease in pH from 5.35 in the case of 0.3% CCMBE to 5.26 in the case of 1.2% CCMBE. The decrease in pH in cream cheese samples with the addition of MBE occurs as a result of the gradual release of phenolic acids from BE, a fact also confirmed by the research carried out by Hala et al. [50]. 

Subsequently, during the storage time, the pH of both the CC sample and the cream cheese with MBE decreased gradually. Although the initial pH of the CC sample was higher than that of the CCMBE samples, at the end of storage, the pH was lower than that of the rest of the samples. On the 28th day of storage, the pH values of the cream cheese varied between 5.12 (CC) and 5.20 (1.2% CCMBE). The addition of MBE in cream cheese samples from 0.6 to 1.2% inhibited the post-fermentation process during storage, the pH decrease being more evident in CC. Therefore, MBE prevents the development of microorganisms during storage, which demonstrates their preservation potential. Similarly, Weragama et al. [3] observed that untreated cream cheese showed lower pH values compared to samples of cream cheeses fortified with powder from dried curry leaves (*Murraya koenigii* L.) during storage. Azarashkan et al. [49] reported that the pH value of the samples of ricotta cheese with nano-encapsulated broccoli sprout extract was higher than that of the control. In the study by Balabanova et al. [48], the incorporation of pepper extracts encapsulated in Labneh cheese did not show a significant effect on the pH of the samples.

Texture profile analysis (TPA) is determined by the type of cheese and corresponds to its sensory characteristics [51]. Table 6 shows the texture parameters (hardness, springiness, cohesiveness, adhesiveness, and gumminess) of the cream cheese analyzed during the 28-day storage time at 4 °C.

The chemical composition of cheeses is one of the important factors affecting their textural properties [19]. Another factor that affects the textural properties of cheeses is pH. In this sense, Soodam et al. [51] determined that curd obtained by coagulation under the action of chymosin at pH 6.5 was harder than that obtained at pH 6.1. In this study, increasing the concentration of the MBE addition in cream cheese led to a slight reduction in protein, fat, and pH value, and as a result, decreased hardness and adhesiveness of fortified cream cheese, ranging from 1914.1 g (CC) to 1439.8 g (1.2% CCMBE), and from 2252.0 g⋅s (CC) to 1648.4 g⋅s (1.2% CCMBE), respectively. Along with the increase in MBE concentration in cream cheese, an increase in cohesiveness and gumminess was also found, from 0.462% (CC) to 0.568% (1.2% CCMBE) and from 884.3% (CC) to 1009.9% (1.2% CCMBE). The addition of MBE to the cream cheese did not influence the springiness of the analyzed cream cheese. Similar results were also obtained when fortifying ricotta cheese with nano-encapsulated broccoli sprout extract [49], low-fat cut cheese with nanoemulsion-based edible coatings containing oregano essential oil and mandarin fiber [52], and fresh cheese with microcapsules or nanoemulsions with *Opuntiaoligacantha* [53].

During the 28-day storage, the hardness and adhesiveness of the analyzed cheese cream registered an essential increase, and the cohesiveness and gumminess of the samples gradually decreased, with the exception of the CC sample, which on the 28th day of storage, registered a deterioration of the parameters of texture. The improvement in texture parameters of the fortified cream cheese samples is probably due to the better water-holding capacity in the cream cheese fortified with MBE samples compared to the cream cheese without additions. The texture parameters, except springiness, were influenced by increasing the MBE concentration in the samples. During the 28-day storage time, in the case of the 0.9% CCMBE sample, the hardness and adhesiveness increased from 1891.2 g to 3225.6 g and from 2134.5 g⋅s to 3841.4 g⋅s, respectively. However, cohesiveness and gumminess decreased from 0.534% to 0.247% and from 935.7% to 819.3%, respectively.

In general, the texture parameters of the cream cheese samples with the addition of MBE correlated with their sensory properties (Table 4) and their pH values (Table 5). The addition of 0.6–0.9% MBE in the cream cheese determined the inhibition of the post-fermentation process, the improvement of the degree of water retention, and the textural parameters of the cream cheese, thus contributing to the extension of the shelf life of the cream cheese by 7 days compared to the control sample.

The 1.2% CCMBE sample demonstrated high textural parameters, but from a sensory point of view, it was rated low due to a too-pronounced basil taste. An addition of 0.3% MBE to the cream cheese does not ensure sufficient stability of the product during the 28 days of storage.

Therefore, the sodium-alginate-based microcapsules ensured the stability of the polyphenolic and bioactive compounds of the basil extract, and in this way, led to the controlled release of the functional compounds from the cream cheese during the storage time.

### 2.5. Mathematical Modeling

**Figure 3:**
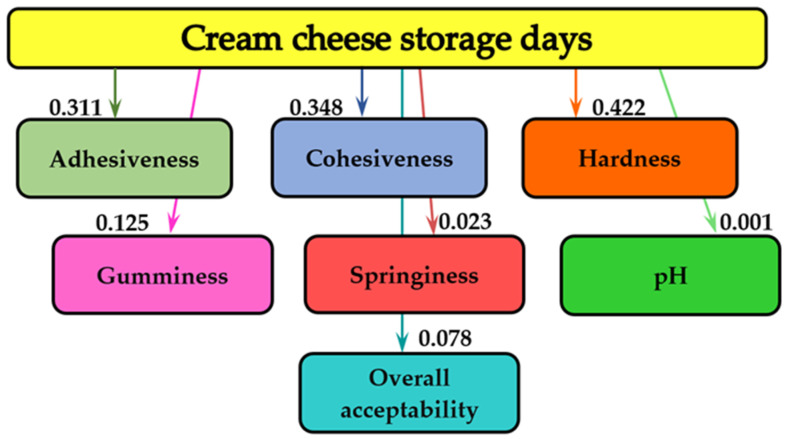
The informational analysis of the influence of storage days on the textural parameters, the pH and on the overall acceptability of cream cheese samples.

The measure of influence of the storage time and the concentrations of MBE that were added to the cream cheese samples on the texture parameters, the pH values, and the sensory analysis (overall acceptability) was evaluated by analysis of mutual information. This mathematical modeling method was also applied in the study of the influence of storage days and the concentration of apple powder on the textural parameters and general acceptability of yogurt for 20 days [54]. The study of the influence of physicochemical quality indicators on the textural characteristics of dry-aged beef for 35 days and different quantities of sea buckthorn and rose hip on the quality of flour products was also evaluated by applying analysis of mutual information [55,56,57]. Figure 3 demonstrates the mutual analysis of the influence of storage days on the textural parameters (adhesiveness, cohesiveness, hardness, gumminess, springiness), the pH, and the overall acceptability of cream cheese samples.

As shown in Figure 3, the storage time of cream cheese samples with different concentrations of MBE additions did not significantly influence the overall acceptability (0.078 bits), the texture parameters (springiness—0.023 bits), and the pH (0.001 bits). The highest information analysis values for texture characteristics were obtained for the hardness (0.422 bits), followed by the cohesiveness (0.348 bits), the adhesiveness (0.311 bits), and the gumminess (0.125 bits).

**Figure 4:**
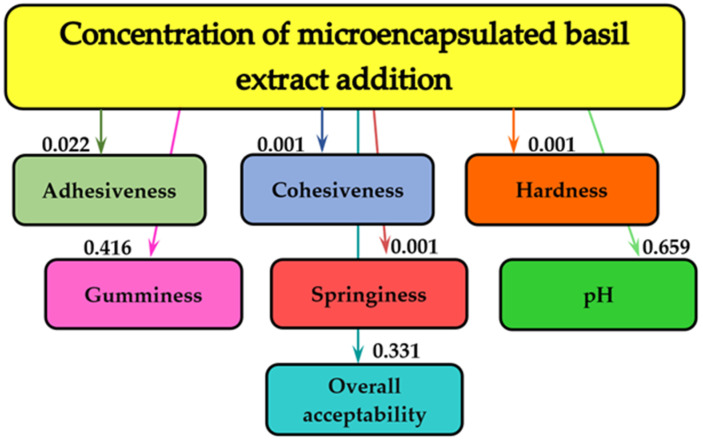
The informational analysis for the influence of the concentration of the MBE added on the textural parameters, the pH, and on the overall acceptability of cream cheese samples.

Figure 4 demonstrates the effects of different concentrations of MBE that were added to the cream cheese samples on the pH, the overall acceptability, and on the textural parameters.

Figure 4 shows that the concentration of the MBE added to the samples influenced the pH (0.659 bits), a textural parameter—gumminess (0.416 bits)—and the overall acceptability of the cream cheese (0.331 bits). In the case of the other textural characteristics (adhesiveness, cohesiveness, hardness, and springiness) the influence of concentration of the MBE was not significant.

## 3. Materials and Methods

### 3.1. Materials

The plant material used in this study consisted of leaves collected from plants of basil (*Ocimum basilicum* L.) that were harvested during August 2022 from AromeNature, Peticeni commune, Calarasi, Republic of Moldova (47°14′10″ N 28°12′31″ E). The basil leaves were dried at 60 ± 1 °C and stored in dark packages at room temperature until extraction. Milk containing 3.8% fat, 3.1% protein, and 4.5% lactose, according to the information on the label, was purchased from JLC, Republic of Moldova. The freeze-dried, direct vat set (FD-DVS) starter culture contains de *Lactococcus lactis* subsp. *lactis* and *Lactococcus lactis* subsp. *cremoris* (CHOOZIT MA 14 LYO, Danisco, Paris, France). Coagulant was used (Marzyme XT 850 IMCU, Danisco, France). Folin–Ciocalteu reagent, sodium carbonate (Na_2_CO_3_) and DPPH (2,2′-diphenyl-picrylhydrazyl), ABTS (2,2′-azino-bis(3-ethylbenzothiazoline-6-sulfonic acid)), Trolox (6-hydroxy-2,5,7,8-tetramethylchroman-2-carboxylic acid), potassium persulfate, calcium chloride, and sodium alginate were purchased from Merck (Darmstadt, Germany), gallic acid equivalent (GAE) were acquired from Sigma-Aldrich (Darmstadt, Germany), and absolute ethanol and methanol were supplied from Chimopar (Bucharest, Romania). Acetonitrile, HPLC-gradient, and the ultrapure water was produced with a Direct-QR 3 UV Water Purification System, Merck (Darmstadt, Germany). The pure standard of chlorogenic acid (>98% HPLC), luteolin (>99% HPLC), gallic acid (>99% HPLC), and cyanidin (>99% HPLC) were purchased from Sigma (Sigma-Aldrich, St. Louis, MO, USA). All reagents used in this study were of analytical grade.

### 3.2. Extraction and Characterization of Basil Extract

#### 3.2.1. Preparation Basil Extract

In order to obtain the hydroalcoholic extract of basil, a mixture of the dry plant and 60% *v*/*v* ethanol in a ratio of 1:10 was sonicated in an Elma Ultrasonic bath (Transsonic T 310 at 35 kHz and an installed power of 95 W) for 30 min at room temperature. Finally, the mixture was centrifuged for 10 min at 7000 rpm, filtered, and stored at 4 °C until further analysis.

#### 3.2.2. Total Polyphenol Content

The obtained extract was characterized in terms of the TPC according to the Folin-Ciocalteu method [58]. First, 5 mL of double distilled water, 1 mL of extract, and 0.5 mL of Folin-Ciocalteu reagent were added to a 10 mL graduated flask. The mixture was stirred, then left to stand for 3 min, and then 1.5 mL of Na_2_CO_3_ (5 g/L) was added. After filling the flask up to 10 mL with double distilled water, it was kept for 16 min at 50 °C (in a water bath), then allowed to cool to room temperature. After cooling, the absorbance of the mixture was read against the control sample (double distilled water) at 765 nm using a UV-VIS T80 spectrophotometer (PG Instruments Limited, Lutterworth, UK). The calculation of the TPC was made with the help of the standard curve of gallic acid equivalent (GAE), drawn for the interval 0.002–0.8 mg/mL. The results were expressed in mg gallic acid equivalent per g of dried weight (DW) of BE (mg GAE/g DW).

#### 3.2.3. HPLC–DAD-ESI-MS Analysis of Polyphenols

Analysis was carried out using an Agilent HP-1200 liquid chromatograph equipped with a quaternary pump, autosampler, DAD detector, and MS-6110 single quadrupole API-electrospray detector (Agilent Technologies, Santa Clara, CA, USA). The positive ionization mode was applied to detect the phenolic compounds; different fragmentors, in the range 50–100 V, were applied. The column was an Eclipse XDB-C18 (5 μm; 4.5 × 150 mm i.d.) from Agilent. The mobile phase was (A) water acidified by 0.1% acetic acid, and (B) acetonitrile acidified by 0.1% acetic acid. The following multistep linear gradient was applied: start with 5% B for 2 min; from 5% to 90% B in 20 min, hold for 4 min at 90% B, then 6 min to arrive at 5% B. The total time of analysis was 30 min, the flow rate was 0.5 mL/min, and the oven temperature was 25 ± 0.5 °C. Mass spectrometric detection of positively charged ions was performed using the Scan mode. The applied experimental conditions were: gas temperature 350 °C, nitrogen flow 7 L/min, nebulizer pressure 35 psi, capillary voltage 3000 V, fragmentor 100 V, and *m*/*z* 120–1200. Chromatograms were recorded at wavelength λ = 280, 340, and 520 nm, and data acquisition was done with the Agilent ChemStation software. The content of specific polyphenols was determined using a comparison of retention times and peaks, with the ones from the chromatogram of a synthetic mix containing the standards listed in Table 7.

#### 3.2.4. Antioxidant Activity 

The antioxidant activity using the DPPH method [59] was determined by adding 0.001 mL extract to 3.9 mL of the DPPH radical solution (0.005 g/200 mL methanol), to 3.9 mL of methanol (control). The mixtures were left to stand in the dark for 10 min, then the absorbance at 515 nm was read with the UV-VIS T80 spectrophotometer (PG Instruments Limited, Leicestershire, UK). The results were calculated from the Trolox calibration curve (R^2^ = 0.9994), drawn for concentrations in the range of 0.004–3.2 mM, and the results were expressed in mM Trolox/g DW.

The antioxidant activity using the ABTS method [60] was determined by adding 0.34 mL extract to 3.4 mL ABTS solution. The mixtures were left to stand in the dark for 6 min, then the absorbance at 734 nm was read with the UV-VIS T80 spectrophotometer (PG Instruments Limited, Leicestershire, UK). The results were calculated from the Trolox calibration curve (R^2^ = 0.9982), drawn for concentrations in the range of 0.01–0.4 mM, and the results were expressed in mM Trolox/g DW.

#### 3.2.5. Antimicrobial Activity

For this analysis and for the preparation of a microencapsulated basil extract, BE was used from which the alcohol was evaporated in a Heidolph Rotavapor (Heidolph Instruments GmbH & Co, Schwabach, Germany) at a temperature of 40 °C and a pressure of 175 mbar.

##### Test Microorganisms

Microorganisms used for the antimicrobial assay contain gram-positive bacteria *Staphylococcus aureus* ATCC 25923, *Bacillus cereus* ATCC 11778, *Enterococcus faecalis* ATCC 19433, and *Geobacillus stearothermophilus* ATCC 7953, gram-negative bacteria *Escherichia coli* ATCC 25922, *Acinetobacter baumannii* ATCC^®^ BAA-747, *Salmonella Abony* NCTC 6017, and yeast *Candida albicans* ATCC 10231. Standard bacterial cultures were offered by the Discipline of Microbiology and Immunology, at the Nicolae Testemitanu State University of Medicine and Pharmacy (Chisinau, Republic of Moldova).

##### Agar Well Diffusion Method

For the quality antimicrobial activity screening of the studied biologic compounds, the well method was used, which is standardized for microbial activity control and proposed by the CLSI standard (Clinical and Laboratory Standards Institute). Wells with a diameter of 6 ± 0.1 mm were made on Müeller–Hinton agar plates, and the distances between neighboring wells and to the edge of the plate were equal. The plates were inoculated with a sterile swab moistened with microbial suspension according to the 0.5 Mac Farland turbidity standard. Equal volumes of tested compounds and solutions of the positive control were introduced into the formed wells. To minimize differences caused by the time intervals at which the test compounds were applied, the plates were left at room temperature for 1–2 h, then thermostated at 37 °C for 24–48 h. The total inhibition area diameter for microbial culture growth (including the wells diameter) was measured with the digital caliper at a 0.1 mm accuracy [61,62].

##### Minimal Inhibitory Concentration and Minimum Bactericidal Concentration

The method of two-fold dilution is a quantitative method for determining the MIC, which is the minimum quantitative value capable of suppressing the growth of microorganisms. To determine the MIC, for each derivative, a discontinuous concentration gradient was created in tubes with Müeller–Hinton broth. Then, 100 μL of bacterial suspension corresponding to the 0.5 McFarland turbidity standard was added to each tube. The tubes were incubated at the cardinal temperature for the growth of the tested species. This was followed by the determination of the MIC value by macroscopic examination of the tubes in order to test for the presence or absence of the growth of the tested microorganisms. The lowest concentration in the tube in which the visible growth of the culture was inhibited represents the MIC value (µg/mL) for the tested compound. In the other tubes and in the control tube, the turbidity of the medium is attested as a result of the multiplication of microorganisms. This method also permitted the determination of the MBC value for the tested compound. For the determination of MBC, each dilution was subcultured on Müeller–Hinton agar plates; they were subsequently incubated and the results were determined. The MBC value is given by the lowest concentration of the tested compound that reduces the number of colonies on the plate by up to 99.9%. All assays were performed in triplicate [63].

### 3.3. Preparation and Characterization of Microencapsulated Basil Extract

#### 3.3.1. Preparation Microencapsulated Basil Extract

Microencapsulated basil extract (MBE) in sodium alginate was prepared according to a slightly modified method of Rijo et al. [64]. Thus, a mixture of sodium alginate (0.6 g) and ultrapure water (20 mL) was stirred on a plate at 40 °C for 1 h and 400 rpm. After cooling to room temperature, 10 mL of BE was added with stirring, continuing the stirring for another 10 min. The resulting solution was added to a 0.2 M CaCl_2_ solution using a syringe. The addition of the mixture was carried out under continuous stirring, in a time interval of 20 min. The stirring continued for another 15 min, after which the beads were washed three times with ultrapure water and then lyophilized.

#### 3.3.2. Physicochemical Analysis

The moisture content and solubility was determined according to Nwabor et al. [65]. The swelling index was determined according to Surini et al. [66].

#### 3.3.3. Scanning Electron Microscope

The scanning electron microscope (SEM) Hitachi SU8230 (Hitachi, Tokyo, Japan) was used for the morphological examination of the samples. The microscope was operated at 30 kV, under vacuum conditions. For examination, the samples were coated with a 9 nm layer of Pt–Pd.

#### 3.3.4. Fourier-Transform Infrared Spectroscopy

The FTIR spectra were recorded using a FT/IR-6100 FTIR spectrometer (JASCO International Co., Ltd., Tokyo, Japan) in the 4000 to 400 cm^−1^ spectral range, with 4 cm^−1^ resolution by the KBr pellet technique. Each sample has been dispersed in about 300 mg of anhydrous KBr mixed with an agate mortar. The pellets were obtained by pressing the mixture into an evacuated die. The spectra were collected and analyzed with Jasco Spectra Manager v.2 software.

#### 3.3.5. Encapsulation Efficiency

The encapsulation efficiency of the MBE was determined according to Machado et al. [67]. Accordingly, 2.5 mg of beads and 5 mL of sodium citrate (3% *w*/*v*) were sonicated for 30 min. In the end, it was centrifuged for 30 min at 14,000 rpm and the TPC in the supernatant was determined as described in Section 3.2.2. The encapsulation efficiency (%) was determined using to the following formula:

(1) $$EE=\frac{{TPC}_{BE}-{TPC}_{S}}{{TPC}_{BE}}\times 100,\%$$

where ${TPC}_{BE}$ is the total polyphenol content of BE used for encapsulation, and ${TPC}_{S}$ is the total polyphenol content of supernatant (i.e., non-encapsulated polyphenols).

### 3.4. Preparation and Characterization of Cream Cheese with Microencapsulated Basil Extract

#### 3.4.1. Preparation Cream Cheese with Microencapsulated Basil Extract

Standardized milk with 3.8% fat content was pasteurized at 63–68 °C for 30 min and cooled to 30–32 °C. Later, 5 DCU/100 L milk of mesophilic starter culture CHOOZIT MA 14 LYO and 0.05% *v*/*v* coagulant Marzyme XT 850 IMCU were added to the milk. The milk was mixed and coagulated for 2.0–2.5 h until a firm curd was formed. The curd was cut, mixed, and dehydrated in lavsan bags for 1 h. Afterwards, the cheese was removed from the bags and cooled to 6–10 °C. To obtain the cream cheese, the cheese was pasted with salt (0.7% *w*/*w*) and different levels of MBE (0.3%, 0.6%, 0.9%, and 1.2% *w*/*w*, relative to the cream cheese). Cream cheese samples were stored in sterilized airtight containers at 4 °C until further analysis.

#### 3.4.2. Physicochemical Analysis

The fat content was determined using gravimetric methods [68]. The dry matter content was determined using ISO 5534:2004 [69]. The protein content was determined using Kjeldahl methods [70]. The pH was measured with a Titrator SI Analytics TitroLine^®^ 5000 (Xylem Analytics, Letchworth, UK) at 20 °C. The physicochemical properties, except for pH, were determined only on the first day of storage.

#### 3.4.3. Sensory Analysis

The sensory analysis of the cream cheese samples was determined using the 5-points scoring scale, according to ISO 22935-3:2009 [71], by a panel of 11 assessors who were selected according to ISO 8586:2012 [72]. Appearance, texture, odor, and taste were evaluated. In scoring each property, the numerical discrete interval scale was used, as follows: 5 points—no deviation from the pre-established sensory specification; 4 points—minimal deviation from the pre-established sensory specification; 3 points—noticeable deviation from the pre-established sensory specification; 2 points—considerable deviation from the pre-established sensory specification, and 1 point—very considerable deviation from the pre-established sensory specification. Sensory properties of the cream cheese must correspond to the quality requirements for milk and dairy products [73] (Table 8).

Overall acceptability of the cream cheese samples was expressed by the total score given by the panel of assessors. To calculate the total score, the average scores per sensory property were added and divided by the number of sensory properties. The sensory analysis of the cream cheese samples was determined at different storage times (1, 7, 14, 21, and 28 days).

#### 3.4.4. Texture Profile Analysis

Texture Profile Analysis (TPA) of the cream cheese samples were analyzed with a TA HD Plus C Texture Analyzer (Stable Micro Systems, Godalming, UK), according to Popescu et al. [54]. Textural parameters were determined on the 1st, 7th, 14th, 21th, and 28th days.

### 3.5. Mathematical Modeling

The MATLAB program (MathWorks, Inc., Natick, MA, USA) was applied to the information analysis in order to determine the influence of storage time and concentrations on textural parameters, pH, and general acceptability of cream cheese samples. The names of textural parameters, pH, and overall acceptability are shown in the rectangles of the graph. The mutual information values, measured in bits, are indicated on the graph arrows. The more pronounced the influence of storage time and concentrations of MBE addition on textural parameters, pH, and overall acceptability, the higher the bit value [74].

### 3.6. Statistical Analysis

All calculations were performed using Microsoft Office Excel 2007 (Microsoft, Redmond, WA, USA). Data obtained in this study are presented as mean values ± the standard error of the mean, calculated from three parallel experiments. The comparison of average values was based on the one-way analysis of variance (ANOVA) according to Tukey’s test, at a significance level of *p* ≤ 0.05, using the Staturphics program, Centurion XVI 16.1.17 (Statgraphics Technologies, Inc., The Plains, VA, USA).

## 4. Conclusions

Basil (*Ocimum basilicum* L.) extract was found to have an important content of phenolic compounds, especially methyl-rosmarinate, rosmarinic acid, rosmadial, carnosol, dehydrodiferulic acid, and chicory, as well as flavonoids (luteolin-glucoside, querectin-rutinoside and epigallocatechin). The extract shows important antioxidant activity—644.75 ± 21.37 mM Trolox/g DW (DPPH) —and inhibitory effects against strains of *Staphylococcus aureus*, *Geobacillus stea-rothermophilus*, *Bacillus cereus*, *Candida albicans*, *Enterococcus faecalis*, *Escherichia coli*, and *Salmonella Abony*.

Microencapsulation of BE in sodium alginate allowed us to obtain MBE with the following characteristics: moisture—6.21 ± 0.08%, swelling index—87.4 ± 0.4%, and solubility—22.1± 1.1%. FTIR spectroscopy demonstrated the existence of weak physical interactions between the components. The encapsulation efficiency of MBE was 78.59 ± 0.01%, which shows that the formed microcapsules can serve as polyphenol carriers for food.

Sensory properties of MBE-fortified cream cheese, including appearance, texture, odor, taste, and overall acceptability were evaluated over a 28-day storage time at 4 °C. The optimal concentration of MBE in cream cheese is in the range of 0.6–0.9%.

The addition of MBE initially led to a decrease in pH, but at the end of storage, the pH of the samples with added MBE was higher that of the control samples, which confirms the preservation potential of MBE due to the inhibition of microorganisms.

After 28 days of storage, the hardness and adhesiveness of the cream cheese showed an essential increase, and the cohesiveness and gumminess of the samples gradually decreased. The improvement in texture parameters is probably due to the better water-holding capacity of MBE-fortified cream cheese.

The measure of influence of storage time and concentrations of the MBE that was added to cream cheese samples on texture parameters, pH values, and sensory analysis (overall acceptability) was evaluated by mutual information analysis. In particular, the concentration of MBE influenced the pH, the textural parameter—gumminess—and the overall acceptability of the product.

The addition of 0.6–0.9% MBE in cream cheese inhibited the post-fermentation process, improved the degree of water retention, and improved the textural parameters of the cream cheese, thus contributing to the extension of the shelf life of the product by 7 days compared to the control sample. Microcapsules based on sodium alginate ensured the stability of the polyphenolic compounds of the basil extract and led to their controlled release from the cream cheese during the storage.

## Figures and Tables

**Table 1:** The content of total and individual polyphenols and the antioxidant activity of basil extract used for experiments.

Indices	Quantity
Total Polyphenol Content (Folin–Ciocalteu), mg GAE/g DW	26.18 ± 0.21
Epigallocatechin, mg/g DW	0.72 ± 0.09
Chicoric acid, mg/g DW	1.30 ± 0.13
Querectin-rutinoside, mg/g DW	0.75 ± 0.02
Luteolin-glucoside, mg/g DW	0.85 ± 0.05
Dehydrodiferulic acid, mg/g DW	3.10 ± 0.26
Rosmarinic acid, mg/g DW	13.81 ± 0.57
Methyl-rosmarinate, mg/g DW	17.08 ± 0.39
Carnosol, mg/g DW	4.78 ± 0.06
Rosmadial, mg/g DW	6.45 ± 0.01
Not identified	8.46 ± 0.17
Antioxidant activity (DPPH), mM Trolox/g DW	644.75 ± 21.37
Antioxidant activity (ABTS), mM Trolox/g DW	8.95 ± 0.03

DPPH—2,2-diphenyl-1-picrylhydrazyl; ABTS—2,2′-Azino-bis(3-ethylbenzthiazoline-6-sulfonic acid). Values in the table represent the means of three replicated trials ± standard deviation.

**Table 2:** Antimicrobial activity of the basil extract against bacteria and yeast strains.

Test Strains	Zone of Inhibition (mm) *	Basil Extract, mg/mL	
		MIC	MBC/MFC
Gram-positive bacteria			
*Bacillus cereus*	11.0 ± 0.5 ^b^	22.5 ± 1.5 ^b^	22.5 ± 1.5 ^a^
*Enterococcus faecalis*	8.3 ± 0.5 ^a^	45.0 ± 0.0 ^c^	90.0 ± 0.0 ^c^
*Staphylococcus aureus*	26.3 ± 0.1 ^e^	11.2 ± 0.6 ^a^	45.0 ± 1.3 ^b^
*Geobacillus stearothermophilus*	15.3 ± 0.5 ^d^	11.2 ± 1.0 ^a^	22.5 ± 1.0 ^a^
Gram-negative bacteria			
*Escherichia coli*	8.3 ± 0.5 ^a^	22.5 ± 1.3 ^b^	45.0 ± 1.7 ^b^
*Acinetobacter baumannii*	12.0 ± 0.6 ^b,c^	11.2 ± 0.8 ^a^	45.0 ± 1.5 ^b^
*Salmonella Abony*	8.3 ± 0.4 ^a^	45.0 ± 0.0 ^c^	45.0 ± 2.0 ^b^
Yeast			
*Candida albicans*	11.0 ± 0.7 ^b^	22.5 ± 1.0 ^b^	22.5 ± 1.3 ^a^

* Diameter of inhibition zone; MIC—minimum inhibitory concentration; MBC—minimum bactericidal concentration; MFC—minimum fungicide concentration. Values in the table represent the means of three replicated trials ± standard deviation. Different letters (^a–e^) designate statistically different results (*p* ≤ 0.05).

**Table 3:** Physicochemical parameters of cream cheese fortified with microencapsulated basil extract.

Parameters	Samples				
	CC	0.3% CCMBE	0.6% CCMBE	0.9% CCMBE	1.2% CCMBE
Dry matter, %	34.32 ± 0.02 ^a^	34.50 ± 0.01 ^b^	34.69 ± 0.02 ^c^	34.89 ± 0.03 ^d^	35.09 ± 0.03 ^e^
Protein content, %	5.82 ± 0.0 ^e^	5.78 ± 0.0 ^d^	5.77 ± 0.01 ^c,d^	5.75 ± 0.01 ^b,c^	5.73 ± 0.01 ^a,b^
Fat content, %	23.04 ± 0.0 ^e^	22.93 ± 0.01 ^d^	22.86 ± 0.01 ^c^	22.79 ± 0.01 ^b^	22.72 ± 0.02 ^a^

CC—cream cheese without microencapsulated basil extract; CCMBE—cream cheese with microencapsulated basil extract. Values in the table represent the means of three replicated trials ± standard deviation. Different letters (^a–e^) designate statistically different results (*p* ≤ 0.05).

**Table 4:** Sensory properties’ (score) evolution in cream cheese fortified with microencapsulated basil extract, during storage.

Sensory Properties	Storage Time, Day	Samples				
		CC	0.3% CCMBE	0.6% CCMBE	0.9% CCMBE	1.2% CCMBE
Appearance	1	5.00 ± 0.0 ^b^	5.00 ± 0.0 ^b^	5.00 ± 0.0 ^b^	5.00 ± 0.0 ^b^	5.00 ± 0.0 ^b^
	7	5.00 ± 0.0 ^b^	5.00 ± 0.0 ^b^	5.00 ± 0.0 ^b^	5.00 ± 0.0 ^b^	5.00 ± 0.0 ^b^
	14	5.00 ± 0.0 ^b^	5.00 ± 0.0 ^b^	5.00 ± 0.0 ^b^	5.00 ± 0.0 ^b^	5.00 ± 0.0 ^b^
	21	5.00 ± 0.0 ^b^	5.00 ± 0.0 ^b^	5.00 ± 0.0 ^b^	5.00 ± 0.0 ^b^	5.00 ± 0.0 ^b^
	28	3.53 ± 0.01 ^a^	3.60 ± 0.1 ^a^	5.00 ± 0.0 ^b^	5.00 ± 0.0 ^b^	5.00 ± 0.0 ^b^
Texture	1	5.00 ± 0.0 ^c^	5.00 ± 0.0 ^c^	5.00 ± 0.0 ^c^	5.00 ± 0.0 ^c^	5.00 ± 0.0 ^c^
	7	5.00 ± 0.0 ^c^	5.00 ± 0.0 ^c^	5.00 ± 0.0 ^c^	5.00 ± 0.0 ^c^	5.00 ± 0.0 ^c^
	14	5.00 ± 0.0 ^c^	5.00 ± 0.0 ^c^	5.00 ± 0.0 ^c^	5.00 ± 0.0 ^c^	5.00 ± 0.0 ^c^
	21	5.00 ± 0.0 ^c^	5.00 ± 0.0 ^c^	5.00 ± 0.0 ^c^	5.00 ± 0.0 ^c^	5.00 ± 0.0 ^c^
	28	4.42 ± 0.01 ^a^	4.69 ± 0.01 ^b^	5.00 ± 0.0 ^c^	5.00 ± 0.0 ^c^	5.00 ± 0.0 ^c^
Odor	1	5.00 ± 0.0 ^e^	5.00 ± 0.0 ^e^	5.00 ± 0.0 ^e^	5.00 ± 0.0 ^e^	4.62 ± 0.02 ^d^
	7	5.00 ± 0.0 ^e^	5.00 ± 0.0 ^e^	5.00 ± 0.0 ^e^	5.00 ± 0.0 ^e^	4.50 ± 0.01 ^c^
	14	5.00 ± 0.0 ^e^	5.00 ± 0.0 ^e^	5.00 ± 0.0 ^e^	5.00 ± 0.0 ^e^	4.42 ± 0.01 ^b,c^
	21	5.00 ± 0.0 ^e^	5.00 ± 0.0 ^e^	5.00 ± 0.0 ^e^	5.00 ± 0.0 ^e^	4.12 ± 0.01 ^a^
	28	4.48 ± 0.1 ^b^	4.62 ± 0.01 ^d^	5.00 ± 0.0 ^e^	5.00 ± 0.0 ^e^	4.10 ± 0.01 ^a^
Taste	1	5.00 ± 0.0 ^h^	5.00 ± 0.0 ^h^	5.00 ± 0.0 ^h^	5.00 ± 0.0 ^h^	4.20 ± 0.01 ^e^
	7	5.00 ± 0.0 ^h^	5.00 ± 0.0 ^h^	5.00 ± 0.0 ^h^	5.00 ± 0.0 ^h^	4.18 ± 0.01 ^d,e^
	14	5.00 ± 0.0 ^h^	5.00 ± 0.0 ^h^	5.00 ± 0.0 ^h^	5.00 ± 0.0 ^h^	4.10 ± 0.02 ^d^
	21	4.32 ± 0.01 ^f^	4.56 ± 0.01 ^g^	5.00 ± 0.0 ^h^	5.00 ± 0.0 ^h^	4.00 ± 0.01 ^c^
	28	3.60 ± 0.02 ^a,b^	3.68 ± 0.01 ^b^	5.00 ± 0.0 ^h^	5.00 ± 0.0 ^h^	4.00 ± 0.01 ^c^
Overall acceptance	1	5.00 ± 0.0 ^h^	5.00 ± 0.0 ^h^	5.00 ± 0.0 ^h^	5.00 ± 0.0 ^h^	4.71 ± 0.01 ^d,e^
	7	5.00 ± 0.0 ^h^	5.00 ± 0.0 ^h^	5.00 ± 0.0 ^h^	5.00 ± 0.0 ^h^	4.67 ± 0.01 ^d,e^
	14	5.00 ± 0.0 ^h^	5.00 ± 0.0 ^h^	5.00 ± 0.0 ^h^	5.00 ± 0.0 ^h^	4.63 ± 0.01 ^d^
	21	4.83 ± 0.01 ^f^	4.89 ± 0.01 ^g^	5.00 ± 0.0 ^h^	5.00 ± 0.0 ^h^	4.53 ± 0.01 ^c^
	28	4.01 ± 0.01 ^a^	4.15 ± 0.01 ^b^	5.00 ± 0.0 ^h^	5.00 ± 0.0 ^h^	4.53 ± 0.01 ^c^

Values in the table represent the means of three replicated trials ± standard deviation. Different letters (^a–h^) designate statistically different results (*p* ≤ 0.05).

**Table 5:** pH value evolution of cream cheese fortified with microencapsulated basil extract, during storage.

Storage Time, Days	Samples				
	CC	0.3% CCMBE	0.6% CCMBE	0.9% CCMBE	1.2% CCMBE
1	5.41 ± 0.0 ^h^	5.35 ± 0.01 ^g^	5.31 ± 0.01 ^f^	5.30 ± 0.02 ^e,f^	5.26 ± 0.01 ^d^
7	5.41 ± 0.0 ^h^	5.34 ± 0.01 ^g^	5.30 ± 0.01 ^e,f^	5.28 ± 0.01 ^d,e^	5.24 ± 0.02 ^c,d^
14	5.35 ± 0.01 ^g^	5.31 ± 0.01 ^f^	5.30 ± 0.01 ^e,f^	5.27 ± 0.02 ^d,e^	5.21 ± 0.01 ^b,c^
21	5.24 ± 0.01 ^c,d^	5.27 ± 0.01 ^d,e^	5.28 ± 0.01 ^d,e^	5.26 ± 0.01 ^d^	5.21 ± 0.01 ^b,c^
28	5.12 ± 0.01 ^a^	5.19 ± 0.01 ^b^	5.27 ± 0.01 ^d,e^	5.25 ± 0.01 ^c,d^	5.20 ± 0.01 ^b^

Values in the table represent the means of three replicated trials ± standard deviation. Different letters (^a–h^) designate statistically different results (*p* ≤ 0.05).

**Table 6:** Texture parameters’ evolution of cream cheese fortified with microencapsulated basil extract, during storage.

Texture Parameters	Storage Time, Day	Samples				
		CC	0.3% CCMBE	0.6% CCMBE	0.9% CCMBE	1.2% CCMBE
Hardness, g	1	1914.1 ± 43.5 ^b^	1914.8 ± 34.1 ^b^	1913.5 ± 28.6 ^b^	1891.2 ± 10.2 ^b^	1439.8 ± 42.3 ^a^
	7	2951.8 ± 60.2 ^e^	2568.8 ± 25.2 ^d^	2476.1 ± 25.4 ^d^	2405.1 ± 27.8 ^c^	1960.9 ± 52.3 ^b^
	14	4123.5 ± 32.5 ^h,i^	2807.2 ± 19.7 ^e^	2637.3 ± 32.5 ^d^	2506.9 ± 31.5 ^d^	2034.01 ± 39.6 ^b^
	21	4180.3 ± 22 ^i^	3254.3 ± 26.8 ^f^	2962.4 ± 31.6 ^e^	2960.8 ± 26.8 ^e^	2502.2 ± 44.3 ^d^
	28	2424.6 ± 17.4 ^c^	3512.01 ± 35.6 ^g^	3280.9 ± 18.8 ^f^	3225.6 ± 34.4 ^f^	3016.9 ± 36.8 ^e,f^
Springiness, %	1	1.001 ± 0.0001 ^b^	1.000 ± 0.0001 ^a^	1.000 ± 0.0001 ^a^	1.002 ± 0.0001 ^c^	1.000 ± 0.0001 ^a^
	7	1.000 ± 0.0001 ^a^	1.001 ± 0.0001 ^b^	1.000 ± 0.0001 ^a^	1.001 ± 0.0001 ^b^	1.002 ± 0.0001 ^c^
	14	1.001 ± 0.0001 ^b^	1.001 ± 0.0001 ^b^	1.001 ± 0.0001 ^b^	1.002 ± 0.0001 ^c^	1.000 ± 0.0001 ^a^
	21	1.001 ± 0.0001 ^b^	1.001 ± 0.0001 ^b^	1.001 ± 0.0001 ^b^	1.001 ± 0.0001 ^b^	1.000 ± 0.0001 ^a^
	28	1.001 ± 0.0001 ^b^	1.001 ± 0.0001 ^b^	1.001 ± 0.0001 ^b^	1.001 ± 0.0001 ^b^	1.001 ± 0.0001 ^b^
Cohesiveness, %	1	0.462 ± 0.008 ^h^	0.476 ± 0.004 ^i^	0.489 ± 0.009 ^i^	0.534 ± 0.004 ^j^	0.568 ± 0.002 ^k^
	7	0.214 ± 0.005 ^b,c^	0.295 ± 0.004 ^d^	0.315 ± 0.007 ^e^	0.390 ± 0.004 ^g^	0.386 ± 0.006 ^f,g^
	14	0.148 ± 0.006 ^a^	0.241 ± 0.007 ^c^	0.313 ± 0.009 ^e^	0.360 ± 0.009 ^f^	0.367 ± 0.008 ^f^
	21	0.199 ± 0.007 ^b^	0.204 ± 0.009 ^b^	0.245 ± 0.006 ^c^	0.282 ± 0.006 ^d^	0.307 ± 0.005 ^e^
	28	0.327 ± 0.007 ^e^	0.202 ± 0.005 ^b^	0.210 ± 0.005 ^b,c^	0.247 ± 0.005 ^c^	0.279 ± 0.007 ^d^
Adhesiveness, g⋅s	1	2252.0 ± 26.7 ^c,d^	2217.0 ± 31.2 ^c^	2216.2 ± 29.7 ^c^	2134.5 ± 26.3 ^c^	1648.3 ± 35.4 ^a^
	7	3589.8 ± 30.2 ^h^	3048.8 ± 25.7 ^f^	2914.6 ± 32.3 ^f^	2772.6 ± 34.7 ^e^	2276.0 ± 18.0 ^d^
	14	4833.9 ± 5.2 ^k^	3175.4 ± 34.8 ^g^	3104.8 ± 24.8 ^f,g^	2821.7 ± 29.8 ^e,f^	2336.0 ± 22.4 ^d^
	21	4903.0 ± 18.6 ^k^	3895.1 ± 28.6 ^i^	3606.3 ± 27.5 ^h^	3125.6 ± 25.5 ^f,g^	2841.6 ± 32.6 ^e,f^
	28	1929.6 ± 35.6 ^b^	4079.9 ± 31.9 ^j^	3916.5 ± 31.7 ^i^	3841.4 ± 31.3 ^i^	3318.4 ± 25.1 ^g^
Gumminess, %	1	884.3 ± 6.7 ^i^	888.8 ± 10.2 ^i^	897.8 ± 9.7 ^i^	935.7 ± 10.9 ^j^	1009.9 ± 7.6 ^l^
	7	631.7 ± 8.5 ^a,b^	756.9 ± 11.3 ^e^	757.8 ± 9.6 ^e^	927.2 ± 9.6 ^j^	980.0 ± 11.5 ^k,l^
	14	618.5 ± 8.6 ^a,b^	673.7 ± 9.1 ^c^	752.6 ± 9.4 ^e^	902.5 ± 11.7 ^i,j^	923.7± 10.5 ^j^
	21	611.9 ± 9.2 ^a^	663.9 ± 10.6 ^b,c^	725.8 ± 11.5 ^d,e^	834.9 ± 9.2 ^g,h^	899.1± 11.3 ^i^
	28	792.8 ± 10.1 ^f^	634.9 ± 11.9 ^a,b^	717.1 ± 10.1 ^d^	819.3 ± 10.3 ^g^	843.4± 12.4 ^g,h^

Values in the table represent the means of three replicated trials ± standard deviation. Different letters (^a–l^) designate statistically different results (*p* ≤ 0.05).

**Table 7:** Polyphenols used as standards in HPLC analysis of basil extract.

Compound	Max Absorbtion, nm	Retention Time, min	*m*/*z* [M+H]^+^	Polyphenol Classes
Epigallocatechin	280	13.17	306	Flavavol
Chicoric acid	330	14.38	475	Hydroxycinnamic acid
Querectin-rutinoside	355, 260	15.73	611	Flavonol
Luteolin-glucoside	350, 260	16.14	449	Flavone
Dehydrodiferulic acid	330	19.34	386, 194	Hydroxycinnamic acid
Rosmarinic acid	330	20.09	360	Hydroxycinnamic acid
Methyl-rosmarinate	330	22.39	375	Hydroxycinnamic acid
Carnosol	330, 270	23.17	331	Phenolic terpene
Rosmadial	330	23.56	345	Phenolic terpene
Not identified	270	24.75	624, 249	

**Table 8:** Sensory characteristics of the cream cheese.

Sensory Properties	Description
Appearance	Soft, creamy, clean, without curdling, white to slightly yellow color in the case of plain cream cheese, or characteristic of the added ingredients in the case of flavored cream cheese.
Texture	Fine, creamy paste
Odor	Characteristic odor of lactic fermentation in the case of plain cream cheese, and the specific odor of the used ingredients in the case of flavored cream cheese, without any foreign odor.
Taste	Characteristic of lactic fermentation in the case of plain cream cheese, or specific to the used ingredients in the case of flavored cream cheese, without any foreign taste.

## Data Availability

No new data were created or analyzed in this study. Data sharing is not applicable to this article.

## Sample Availability

Not available.
